# Comparative Morphological and Proteomic Characterization of Adipose Tissues from Different Anatomical Sites in Yili Horses

**DOI:** 10.3390/biology15080621

**Published:** 2026-04-16

**Authors:** Liping Yang, Lirong Song, Zhixin Lu, Xinkui Yao, Jianwen Wang, Yaqi Zeng, Wanlu Ren, Penghui Luo, Jun Meng

**Affiliations:** 1 College of Animal Science, Xinjiang Agricultural University, Urumqi 830052, China; yangliping9817@126.com (L.Y.); 18299515075@163.com (L.S.); 18199821647@163.com (Z.L.); yxk61@126.com (X.Y.); wjw1262022@126.com (J.W.); xjauzengyaqi@163.com (Y.Z.); 13201295117@163.com (W.R.); 2 Xinjiang Key Laboratory of Horse Breeding and Sports Physiology, Xinjiang Agricultural University, Urumqi 830052, China; 3 Horse Industry Research Institute, Xinjiang Agricultural University, Urumqi 830052, China; 4 Xinjiang Uygur Autonomous Region Animal Husbandry Station, Urumqi 830004, China; xmzztxzx1@163.com

**Keywords:** Yili horses, subcutaneous adipose tissue, pericardial adipose tissue, adipocyte morphology, proteomics

## Abstract

Adipose tissue functions not only as a primary energy reservoir but also as a metabolically active endocrine organ. However, the morphological and molecular differences among adipose depots from different anatomical sites in Yili horses remain unclear. This study aimed to compare the morphological characteristics and proteomic profiles of subcutaneous adipose tissue (SAT) and pericardial adipose tissue (PCAT). To this end, adipose tissue samples from 18 Yili horses were analyzed using hematoxylin and eosin (H&E) staining, while a subset of samples from 6 horses was subjected to proteomic analysis. The results demonstrated that adipocytes in PCAT showed significantly larger areas and diameters but a lower number per field than those in SAT (*p* < 0.01). Proteomic profiling identified 451 differentially expressed proteins (DEPs) between SAT and PCAT. Gene Ontology (GO) and Kyoto Encyclopedia of Genes and Genomes (KEGG) pathway enrichment analyses indicated that these DEPs were primarily involved in fatty acid catabolism, glycolysis, ECM–receptor interaction, thermogenesis, Wnt signaling, and other related pathways. Notably, enrichment analyses further revealed that SAT exhibited more active substrate utilization, energy metabolism, and lipid turnover, whereas PCAT was more associated with structural regulation and cardiovascular-related signaling pathways. Furthermore, correlation analysis between adipocyte morphological metrics and proteomic data identified ACAA2, ENO1, TPI1, PLIN1, COL6A3, and ITGB1 as candidate proteins regulating the site-specific differences in morphology and metabolic function between SAT and PCAT. These findings reveal distinct morphological and proteomic features of different adipose depots in Yili horses, providing a foundation for understanding depot-specific adipose function and its underlying regulatory mechanisms.

## Simple Summary

Adipose tissue is not only an energy storage organ but also plays a key role in regulating metabolism through the secretion of bioactive molecules. Different adipose depots can exhibit distinct structural and functional characteristics, but these differences have not been well characterized in Yili horses. In this study, we compared subcutaneous adipose tissue (SAT) and pericardial adipose tissue (PCAT) to investigate their structural features and functional differences. We found that adipocytes in PCAT were larger but fewer in number than those in SAT. In addition, the two depots showed distinct protein expression patterns, mainly associated with energy metabolism, lipid utilization, and tissue structure. Several proteins were identified as potential regulators of adipocyte size and metabolic activity. These findings enhance our understanding of depot-specific adipose function in horses and provide useful insights for future research on animal metabolism, health, and livestock production.

## 1. Introduction

In addition to their traditional roles in racing, labor, and leisure, horses also serve as important sources of meat and milk in certain regions, particularly in Central Asia [1]. Horse meat has attracted increasing attention due to its favorable nutritional profile, characterized by high protein content, low fat levels, and abundant essential minerals such as iron and zinc [2,3]. In Western European countries, it is regarded as a highly valuable food resource [4,5]. The distribution and composition of adipose tissue play a crucial role in determining meat quality. Specifically, subcutaneous adipose tissue (SAT) and intramuscular fat (IMF) interact with muscle fibers via structural and metabolic mechanisms, thereby affecting meat tenderness, juiciness, and flavor [6]. Han et al. [7] reported that the PPAR and AMPK signaling pathways are involved in IMF deposition in pigs, which in turn affects meat flavor, juiciness, and tenderness.

Adipose tissue, composed mainly of adipocytes derived from preadipocyte differentiation, plays a central role in systemic energy balance and metabolism [8,9]. As an important endocrine organ, it secretes various adipokines that regulate glucose and lipid metabolism, angiogenesis, immune responses, and reproduction [10]. Based on anatomical distribution, adipose tissue can be classified into subcutaneous, visceral, intermuscular, and intramuscular depots. Among these, fat is preferentially stored in SAT, whereas excess lipid accumulation leads to ectopic deposition in organs such as the heart, liver, and skeletal muscle, contributing to metabolic disorders [11]. Different adipose depots exhibit distinct morphological and metabolic characteristics. For example, adipocytes in SAT are generally larger than those in visceral or intramuscular depots, as reported in buffalo and cattle [12,13]. Proteomic analysis provides a powerful approach to linking protein expression with biological function and metabolic phenotypes [14]. Previous proteomic studies have identified key proteins involved in site-specific fat deposition, including NDUFA6, NDUFA9, and ACO2 in pigs [15], as well as SRSF10, CSRP3, and CRTC2 in goat intramuscular adipose tissue [16]. However, most studies have focused on pigs and goats. As a monogastric herbivore, the horse exhibits distinct digestive physiology that may influence adipose tissue metabolism and development. In particular, the Yili horse possesses unique metabolic characteristics, yet proteomic studies across different adipose depots in this species remain limited.

The Yili horse, an indigenous breed developed in Xinjiang, China, is primarily distributed in the Yili and Altay regions. This breed is well adapted to high-altitude, cold environments and exhibits strong endurance capacity [17]. Therefore, adipose tissue in Yili horses functions not only as an energy reservoir but also as an active metabolic tissue supporting environmental adaptation and sustained exercise. Adipose tissue exhibits pronounced functional heterogeneity across anatomical depots: SAT primarily contributes to thermal insulation and energy storage, whereas pericardial adipose tissue (PCAT) plays an important role in local cardiac metabolic regulation [18,19]. Consequently, systematic characterization of the morphological and molecular features of different adipose depots in Yili horses is essential for understanding depot-specific functions and metabolic regulatory mechanisms.

However, despite the recognized heterogeneity of adipose tissue, the molecular mechanisms underlying depot-specific differences in horses, particularly between SAT and PCAT, remain largely unexplored. Comparing anatomically and functionally distinct adipose depots provides a powerful approach to uncover depot-specific regulatory mechanisms and identify key proteins and pathways underlying their specialized functions. Previous studies on adipose tissue heterogeneity have predominantly focused on transcriptomic or metabolomic analyses. In contrast, proteomics, as a technique that directly reflects functional effects, can more accurately reveal depot-specific differences in adipose tissue. The genome-wide correlation between mRNA expression and protein abundance is relatively low, with only approximately 40% of protein expression differences being explained by transcriptional variation [20]; therefore, investigations at the protein level are of greater biological relevance. Data-independent acquisition (DIA) mass spectrometry has emerged in recent years as a high-throughput approach capable of comprehensive and unbiased detection of all peptide ions within a sample, surpassing the limitations of traditional data-dependent acquisition (DDA) strategies [21]. In this study, we employed Astral-DIA quantitative proteomics to systematically compare SAT and PCAT in Yili horses, focusing on differences in lipid metabolism, energy metabolism, structural maintenance, and adipocyte formation. This approach aimed to identify key functional proteins and signaling pathways underlying depot-specific differences. Ultimately, this study provides new insights into the molecular basis of adipose tissue heterogeneity and physiological adaptation in horses, with potential implications for meat quality improvement and horse breeding.

## 2. Materials and Methods

### 2.1. Experimental Animals and Sample Collection

Eighteen clinically healthy male Yili horses (5–6 years old; 423 ± 19 kg, CV = 4.49%) were included in this study. All animals originated from the same grazing population in Zhaosu County, Xinjiang, China, and were managed under comparable conditions. The horses were used for traditional grazing management and were not subjected to racing or intensive exercise. Sampling was conducted in the same season (winter) to minimize seasonal effects on adipose tissue metabolism. The sampling area has a temperate continental climate, characterized by cold and dry winters with average temperatures ranging from −15 °C to −10 °C. Prior to slaughter, animals were fasted for 24 h with free access to water, and their health status was confirmed by veterinary examination to exclude any clinical abnormalities. Slaughter was performed in accordance with standardized industrial protocols and ethical guidelines, and tissue samples were collected immediately post-mortem. From each horse (*n* = 18), approximately 10 g of SAT was excised from the dorsal withers, and approximately 10 g of PCAT was collected from the coronary (atrioventricular) groove surrounding the base of the heart. All samples were rinsed with phosphate-buffered saline (PBS) to remove residual blood and debris. Subsequently, approximately 5 g of each tissue was cut into blocks (1 cm × 1 cm × 0.5 cm) and fixed in 4% paraformaldehyde for histological sectioning, while the remaining approximately 5 g was snap-frozen in liquid nitrogen and stored at −80 °C for subsequent proteomic analysis.

### 2.2. Hematoxylin and Eosin (H&E) Staining

Adipose tissue samples (*n* = 18) fixed in 4% paraformaldehyde were retrieved, dehydrated, and cleared before being embedded in paraffin. Sections were prepared, deparaffinized, and stained with H&E following standard protocols. The stained sections were examined under an optical microscope, and images were captured. For each sample, six non-overlapping fields of view were randomly selected for analysis under consistent criteria. Regions with intact and clearly distinguishable adipocytes were included, whereas areas containing artifacts or non-adipose structures were excluded. Image-Pro Plus 6.0 software (Media Cybernetics, Rockville, MD, USA) was used to measure adipocyte diameter and area, as well as to count the number of adipocytes within each field.

### 2.3. Proteomic Analysis

#### 2.3.1. Protein Extraction and Peptide Digestion

Six horses were randomly selected from the total of 18 using a randomization procedure for proteomic analysis. Adipose tissue samples were homogenized using an MP FastPrep-24 homogenizer (24 × 2, 6.0 m/s, 60 s, twice, MP Biomedicals, Irvine, CA, USA) at low temperature and collected into liquid-nitrogen-precooled centrifuge tubes. Proteins were extracted using sodium dodecyl sulfate-Tris (SDT) buffer (4% SDS, 100 mM Tris-HCl, pH = 7.6), boiled for 15 min, and centrifuged at 14,000× *g* for 40 min at 4 °C. Protein concentration was determined using a BCA Protein Assay Kit (Thermo Fisher Scientific, Waltham, MA, USA). For quality control, 15 µg of protein from each sample was mixed with 5 × loading buffer, boiled for 5 min, separated on 4–20% SDS-PAGE precast gradient gels (180 V constant voltage, 45 min; Bio-Rad Laboratories, Hercules, CA, USA), and visualized with Coomassie Brilliant Blue R-250 staining (Sigma-Aldrich, St. Louis, MO, USA). Reduction using 40 mM dithiothreitol (DTT) was carried out at 37 °C for 1.5 h, followed by alkylation with 20 mM iodoacetamide (IAA) for 30 min at room temperature in the dark. Proteins were digested with trypsin at an enzyme-to-protein ratio of 1:50 (*w*/*w*) overnight at 37 °C using the Filter-Aided Sample Preparation (FASP) method as previously described [22], with Microcon 10 kDa centrifugal filter units (Millipore, Burlington, MA, USA). The resulting peptides were desalted on Empore™ SPE C18 cartridges (30 µm; Waters Corporation, Milford, MA, USA), dried in a vacuum centrifuge, and reconstituted in 40 µL of 0.1% (*v*/*v*) formic acid (LC-MS grade, Fisher Scientific, Waltham, MA, USA). Peptide concentrations were measured spectrophotometrically at 280 nm using a NanoDrop spectrophotometer (Thermo Fisher Scientific, Waltham, MA, USA). Indexed retention time (iRT) standard peptides (Biognosys AG, Schlieren, Switzerland) were spiked into each sample before LC–MS/MS analysis.

#### 2.3.2. DIA Mass Spectrometry Analysis

Peptides from each sample were analyzed using an Orbitrap Astral mass spectrometer (Thermo Fisher Scientific, Waltham, MA, USA) coupled to a Vanquish Neo LC system (Thermo Fisher Scientific, Waltham, MA, USA) operated in DIA mode. Separation was performed on a Thermo Scientific EASY-Spray C18 analytical column (15 cm × 150 µm ID, 2 µm particle size; ES906) under nano-flow conditions using mobile phase A (0.1% formic acid in water) and mobile phase B (0.1% formic acid in 80% acetonitrile). Peptides were eluted with a linear gradient of solvent B prior to mass-spectrometric analysis. In MS1 scans, precursor ions were acquired over the *m*/*z* range 380–980 at a resolution of 240,000 at 200 *m*/*z*, with a normalized automatic gain control (AGC) target of 500% and a maximum injection time of 5 ms. For MS2 spectra in DIA mode, 299 isolation windows (2 *m*/*z* width) were used with higher-energy collisional dissociation (HCD) at a collision energy of 25 eV, a normalized AGC target of 500%, and a maximum injection time of 3 ms.

#### 2.3.3. Mass Spectrometry Data Analysis

DIA data were analyzed using DIA-NN (version 1.8.1, https://github.com/vdemichev/DiaNN, accessed on 17 August 2025). The search enzyme was specified as trypsin, allowing for one missed cleavage. Carbamidomethylation of cysteine was set as a fixed modification, whereas oxidation of methionine and acetylation at the protein N-terminus were set as variable modifications. Protein identifications were validated at a false discovery rate (FDR) of 1% at both peptide and protein levels, corresponding to 99% confidence.

#### 2.3.4. Bioinformatics Analysis

Quantitative data for the identified proteins were subjected to filtering, normalization, and missing-value imputation. Differentially expressed proteins (DEPs) between the two adipose tissue depots were identified based on the criteria of a fold change (FC) > 1.5 or <0.67 and an adjusted *p*-value < 0.05, where *p*-values were corrected using the Benjamini–Hochberg procedure for multiple-testing adjustment. Gene Ontology (GO) enrichment analysis was performed using Blast2GO (version 2.8.0+), and Kyoto Encyclopedia of Genes and Genomes (KEGG) pathway annotation was conducted using KOBAS (KOBAS 3.0, http://bioinfo.org/kobas, accessed on 28 August 2025). Adjusted *p*-values < 0.05 were considered statistically significant and indicative of pathways relevant to adipose tissue heterogeneity. Protein–protein interaction (PPI) networks were constructed and analyzed based on the STRING database (Search Tool for the Retrieval of Interacting Genes/Proteins; http://string-db.org/; accessed on 10 September 2025).

### 2.4. Statistical Analysis

Morphological data for SAT and PCAT were organized using Microsoft Excel. Prior to statistical analysis, data normality was assessed using the Shapiro–Wilk test, and homogeneity of variances was evaluated using Levene’s test. Statistical differences in adipocyte diameter, area, and number were analyzed using the independent samples *t*-test in SPSS 26.0 software (IBM Corp., Armonk, NY, USA). All data are presented as mean ± standard deviation (SD). Graphs were generated using GraphPad Prism software (version 10.1.2, GraphPad Software, San Diego, CA, USA).

## 3. Results

### 3.1. Morphological Differences Between SAT and PCAT in Yili Horses

**Figure 1:**
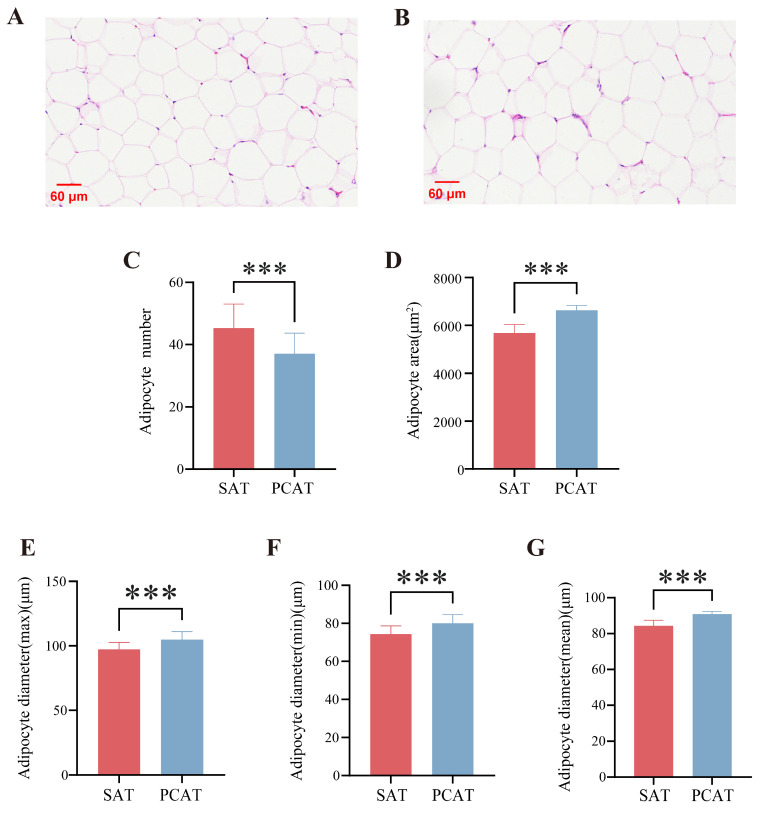
Morphological analysis of subcutaneous and pericardial adipose tissues in Yili horses. (**A**) subcutaneous adipose tissue (SAT); (**B**) pericardial adipose tissue (PCAT); (**C**) Adipocyte number; (**D**) Adipocyte area; and (**E**–**G**) Adipocyte diameter (maximum, minimum, and mean). *** indicates *p* < 0.001.

The H&E staining results of SAT and PCAT from Yili horses are presented in Figure 1. As shown in Figure 1A,B, the adipose tissue exhibited a typical honeycomb-like structure, characterized by large adipocytes with nuclei displaced to the periphery. Blood vessels and connective tissue were also observed distributed within the adipose tissue matrix. Specifically, adipocytes in SAT appeared smaller, compact, and structurally intact, exhibiting polygonal, round, or oval shapes. In contrast, adipocytes in PCAT were larger and more loosely arranged, though they maintained a polygonal morphology. Statistical analysis (Figure 1C–G) revealed significant quantitative differences between the two adipose depots. The number of adipocytes per field was significantly higher in SAT compared to PCAT (*p* < 0.01). Conversely, the adipocyte area, maximum diameter, minimum diameter, and mean diameter in PCAT were all significantly greater than those in SAT (*p* < 0.01).

### 3.2. Proteomic Analysis of SAT and PCAT in Yili Horses

#### 3.2.1. Identification and Quantification of Proteins in SAT and PCAT

**Figure 2:**
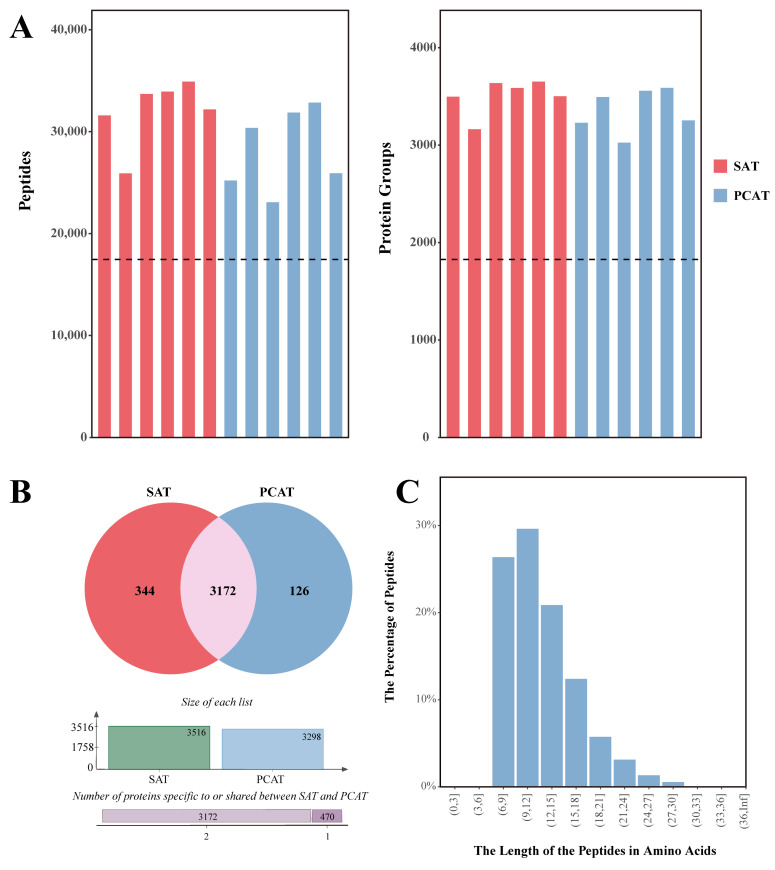
Identification and quantification of proteins in SAT and PCAT from Yili horses. (**A**) Number of proteins and peptides identified in each sample. (**B**) Venn diagram of proteins in the two adipose tissues. (**C**) Distribution of peptide lengths.

In this study, a total of 4085 proteins and 41,937 peptides were identified (Figure 2A). Among these, 344 proteins were uniquely identified in SAT, 126 proteins were uniquely identified in PCAT, and 3172 proteins were commonly identified in both depots (Figure 2B). Peptide length distribution analysis showed that the majority of peptides ranged from 7 to 23 amino acids, which aligns with the cleavage specificity of trypsin, indicating high-quality sample preparation (Figure 2C). Prior to quantitative analysis, raw data underwent filtering, median normalization, and missing value imputation (using the minimum value method) to eliminate systematic bias. This process yielded 3398 valid proteins for subsequent statistical analysis. There were no significant differences in the number of identified proteins (SAT: 3162–3652; PCAT: 3025–3588) or peptides across samples. Furthermore, 2329 proteins were consistently identified across all 12 samples (including 6 SAT samples and 6 PCAT samples), demonstrating the reliable coverage depth and reproducibility of the proteomic data.

#### 3.2.2. Principal Component Analysis (PCA) and Correlation Analysis

**Figure 3:**
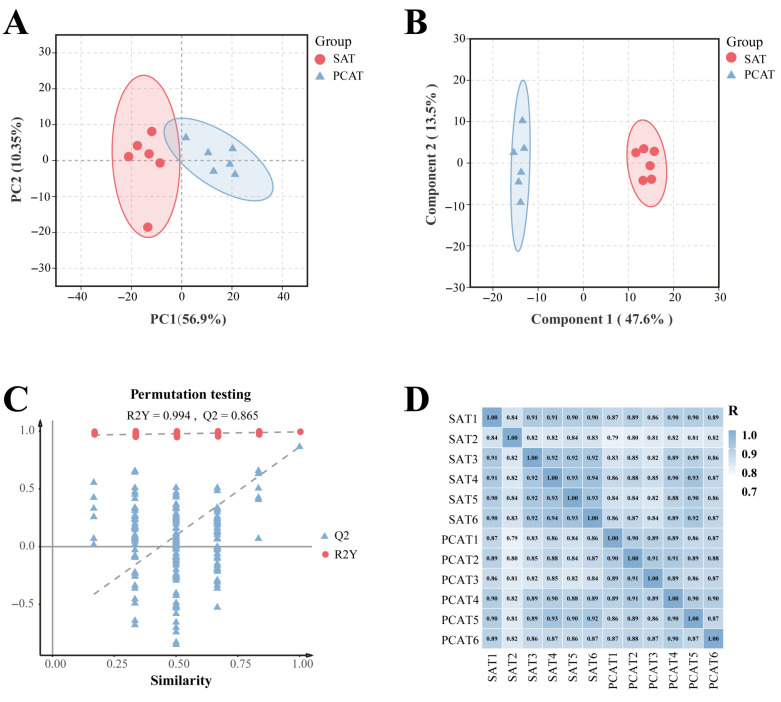
Sample correlation and clustering analyses of proteomic data. (**A**) Principal component analysis (PCA) score plot; (**B**) Orthogonal partial least squares discriminant analysis (OPLS-DA) score plot; (**C**) Validation of the OPLS-DA model. The dashed lines represent the regression lines of R^2^Y and Q^2^ values obtained from the permutation test; and (**D**) Pearson correlation heatmap among samples.

To ensure the reliability and reproducibility of the proteomic data, PCA and correlation analysis were performed. The PCA results revealed that the first principal component (PC1) and the second principal component (PC2) accounted for 56.90% and 10.35% of the variance, respectively. The cumulative contribution rate reached 67.25%, effectively capturing the primary information of the samples (Figure 3A). Building upon the PCA, Orthogonal Partial Least Squares Discriminant Analysis (OPLS-DA) was conducted. As shown in Figure 3B,C, samples from the SAT and PCAT groups exhibited distinct spatial separation between groups and tight clustering within groups. The model parameters (*R*^2^*Y* = 0.99, *Q*^2^ = 0.87) indicated high reliability and predictive capability, confirming significant differences in protein composition between the two adipose depots. Furthermore, the Pearson correlation coefficients between all samples were consistently greater than 0.79, approaching 1.00 (Figure 3D), which indicates high consistency among biological replicates used for proteomic sequencing. In summary, these findings demonstrate significant proteomic heterogeneity between SAT and PCAT, warranting further investigation.

#### 3.2.3. Analysis of DEPs

**Figure 4:**
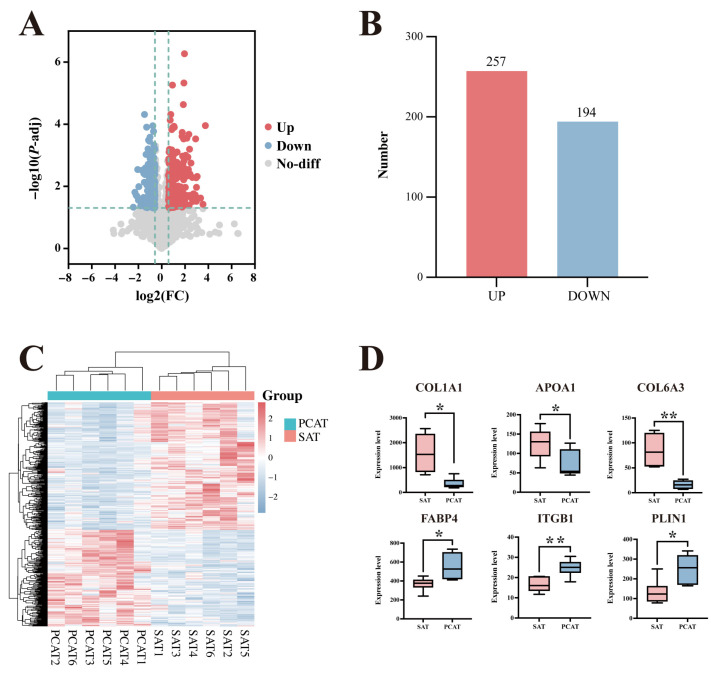
Differential proteomic analysis of SAT and PCAT in Yili horses. (**A**) Volcano plot of differentially expressed proteins (DEPs) between SAT and PCAT. The dashed vertical lines indicate the log_2_FC thresholds, and the dashed horizontal line indicates the significance threshold corresponding to an *p* value of 0.05; (**B**) Statistics of up- and down-regulated proteins; (**C**) Clustering heatmap of DEPs between SAT and PCAT; and (**D**) Boxplots showing quantitative differences in representative DEPs. * indicates *p* < 0.05, and ** indicates *p* < 0.01.

Based on the proteomic sequencing data of SAT and PCAT, a total of 451 DEPs were screened from the 3398 valid proteins. Although the total number of identified proteins was comparable between SAT and PCAT, marked differences in protein expression levels were observed, as reflected by the identified DEPs. Specifically, compared to PCAT, 257 proteins were significantly upregulated and 194 proteins were significantly downregulated in SAT (Figure 4A,B). Hierarchical clustering analysis of the DEPs revealed distinct group separation and contrasting regulation patterns between the two tissue types. Furthermore, samples within the same group clustered tightly, indicating good biological reproducibility and supporting the reliability of the DEP screening results (Figure 4C). To further visualize specific expression differences, boxplots were generated for three representative upregulated and three downregulated proteins. As shown in Figure 4D, the relative abundance of COL1A1, APOA1, and COL6A3 was significantly higher in SAT than in PCAT, whereas the levels of FABP4, ITGB1, and PLIN1 were significantly lower in SAT compared to PCAT.

#### 3.2.4. GO and KEGG Enrichment Analysis of DEPs

To further investigate the functions, cellular localization, and molecular mechanisms of DEPs between SAT and PCAT in Yili horses, GO and KEGG enrichment analyses were performed separately for upregulated and downregulated DEPs.

**Figure 5:**
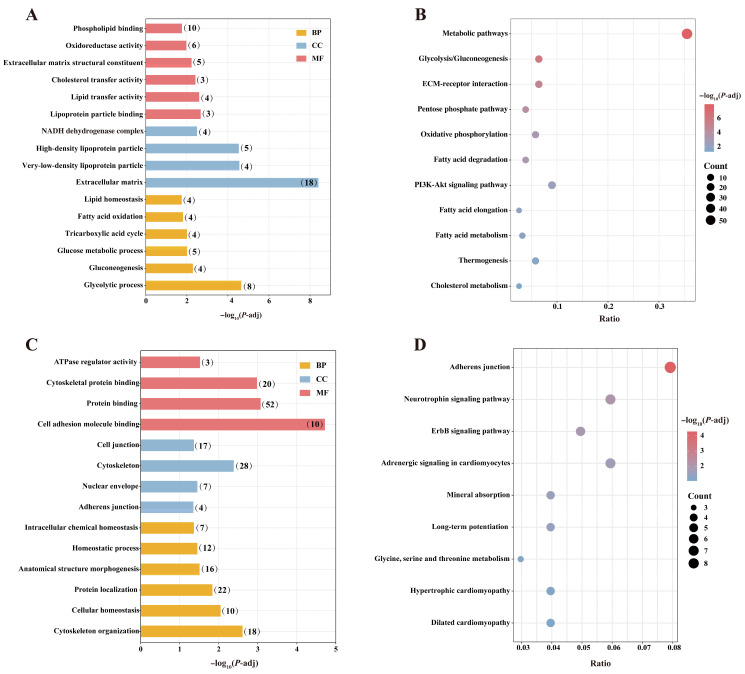
GO and KEGG enrichment analysis of DEPs. (**A**) GO terms enriched for upregulated DEPs. Numbers in brackets indicate the number of proteins enriched in each GO term. (**B**) KEGG pathways enriched for upregulated DEPs. (**C**) GO terms enriched for downregulated DEPs. (**D**) KEGG pathways enriched for downregulated DEPs.

A total of 257 DEPs were significantly upregulated in SAT compared with PCAT, and these proteins were enriched in 692 GO terms and 44 KEGG pathways (*p*-adj < 0.05). These proteins were primarily associated with GO terms related to energy and lipid metabolism, including the glycolytic process, gluconeogenesis, glucose metabolic process, tricarboxylic acid cycle, fatty acid oxidation, lipid homeostasis and lipid transfer activity (Figure 5A). KEGG pathway analysis revealed significant enrichment in metabolic pathways, glycolysis/gluconeogenesis, pentose phosphate pathway, oxidative phosphorylation, fatty acid degradation, fatty acid elongation, fatty acid metabolism, and thermogenesis, among which metabolic pathways contained the largest number of DEPs (Count = 55) (Figure 5B). These findings suggest that upregulated DEPs may contribute to functional differences between SAT and PCAT in substrate utilization, energy metabolism, and lipid synthesis and degradation.

In contrast, 194 DEPs were significantly downregulated in SAT compared with PCAT, and these proteins were enriched in 165 GO terms and 16 KEGG pathways (*p*-adj < 0.05). These proteins were mainly associated with cytoskeleton organization, cellular homeostasis, cell junctions, protein localization, cell adhesion molecule binding, and protein binding, indicating roles in structural maintenance, homeostatic regulation, and protein function (Figure 5C). KEGG enrichment analysis further showed that these DEPs were involved in pathways such as adherens junction, ErbB signaling pathway, adrenergic signaling in cardiomyocytes, hypertrophic cardiomyopathy, and dilated cardiomyopathy (Figure 5D). These results suggest that downregulated DEPs may be involved in functional differences between SAT and PCAT in cellular structural integrity, cell–cell interactions, and receptor-mediated signaling pathways.

Taken together, GO and KEGG enrichment analyses indicate that, compared with PCAT, SAT exhibits more active substrate utilization, energy metabolism, and lipid turnover, whereas PCAT shows greater enrichment in structural regulation and cardiovascular-related signaling pathways. These findings highlight distinct differences between the two adipose depots in metabolic characteristics and local tissue regulatory functions.

#### 3.2.5. PPI Network Analysis of DEPs

To further investigate the interaction mechanisms of DEPs between SAT and PCAT, PPI networks of upregulated and downregulated DEPs were constructed separately using the STRING database. Isolated nodes with low connectivity (fewer than 2 edges) were removed to improve network reliability.

**Figure 6:**
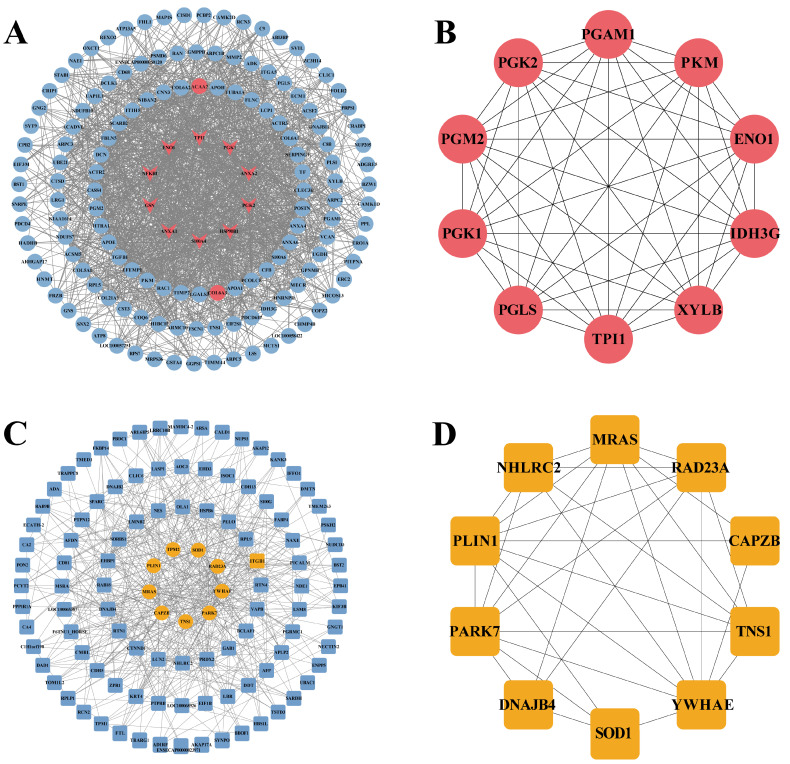
Protein–protein interaction (PPI) analysis of DEPs in adipose tissues. (**A**) PPI network of upregulated DEPs. (**B**) Hub proteins identified from the upregulated DEPs. (**C**) PPI network of downregulated DEPs. (**D**) Hub proteins identified from the downregulated DEPs.

The results showed that 147 upregulated DEPs formed a total of 1608 interaction pairs, among which HSP90B1 (Degree = 56), PGK1 (Degree = 54), ANXA2 (Degree = 53), ENO1 (Degree = 50), TPI1 (Degree = 47), and PGK2 (Degree = 46) exhibited relatively high connectivity (Figure 6A). Further analysis using the Maximal Clique Centrality (MCC) algorithm in the CytoHubba plugin of Cytoscape (version 3.10.0, Cytoscape Consortium, San Diego, CA, USA) identified 10 core candidate proteins among the upregulated DEPs, including PGK2, PGM2, PGK1, PGLS, TPI1, XYLB, IDH3G, ENO1, PKM, and PGAM1 (Figure 6B). In contrast, 109 downregulated DEPs formed 510 interaction pairs, among which YWHAE (Degree = 35), MARS (Degree = 33), PARK7 (Degree = 29), and PLIN1 (Degree = 19) showed relatively high connectivity (Figure 6C). Similarly, 10 core candidate proteins were identified from the downregulated DEPs based on the MCC algorithm, including NHLRC2, MRAS, RAD23A, CAPZB, TNS1, YWHAE, SOD1, DNAJB4, PARK7, and PLIN1 (Figure 6D).

#### 3.2.6. Correlation Analysis of Adipose Morphology and Candidate Proteins in Yili Horses

**Figure 7:**
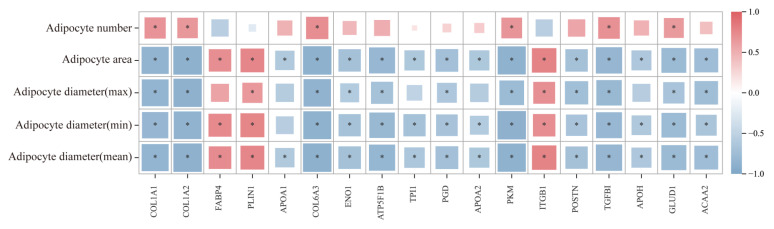
Correlation analysis between adipose tissue morphology and candidate proteins. Red indicates a positive correlation, and blue indicates a negative correlation. * indicates *p* < 0.05.

Correlation analysis revealed significant associations between multiple proteins and adipocyte morphological characteristics (Figure 7). COL1A1, COL1A2, COL6A3, PKM, GLUD1, and TGFBI were significantly positively correlated with adipocyte number, whereas FABP4, PLIN1, and ITGB1 were negatively correlated with adipocyte number. FABP4, PLIN1, and ITGB1 showed significant positive correlations with adipocyte area and diameter, while ENO1, PGD, ACAA2, TPI1, COL6A3, PKM, GLUD1, and TGFBI were significantly negatively correlated with these parameters.

## 4. Discussion

Adipose tissue is a highly complex endocrine organ composed not only of adipocytes but also of various other cell types, including preadipocytes, mesenchymal stem cells, fibroblasts, vascular endothelial cells, T lymphocytes, and macrophages. These cells collectively play essential roles in regulating energy storage, metabolic homeostasis, insulin sensitivity, and systemic inflammation [23]. As the fundamental structural and functional unit of adipose tissue, adipocytes are responsible for energy storage and mobilization, and they exert crucial endocrine functions through the secretion of adipokines such as adiponectin and leptin, which help maintain insulin sensitivity, modulate inflammatory responses, and preserve metabolic balance. The morphology of adipocytes directly reflects regional differences in adipose tissue characteristics, and changes in their size and number are closely associated with the development of metabolic disorders [24]. Previous studies have demonstrated that chronic energy imbalance leads to excessive lipid accumulation within adipocytes, resulting in an increased number of adipocytes and hypertrophy of existing ones [25,26]. Wang et al. reported that the adipocyte area in visceral adipose tissue (VAT) of Kazakh horses was significantly larger than that in abdominal adipose tissue (AAT) and SAT, with AAT showing a significantly greater adipocyte area than SAT but a notably lower cell number [27]. Similarly, Depreester et al. observed that adipocytes in perirenal adipose tissue of Holstein Friesian dairy cows exhibited greater cell area and diameter compared with those in SAT [28]. In this study, SAT in Yili horses was characterized by a smaller adipocyte area and a higher cell number, whereas PCAT exhibited the opposite pattern, with a larger adipocyte area and fewer cells. Moreover, the number of adipocytes in SAT was significantly greater than that in PCAT, whereas the adipocyte area, maximum diameter, minimum diameter, and mean diameter were markedly lower, exhibiting statistically significant differences. These findings are consistent with the results reported in Kazakh horses and Holstein cows, suggesting that site-specific differences in adipocyte morphology may exhibit similar patterns across different animal species. Previous studies have indicated that variations in adipocyte size among different adipose depots are associated with depot-specific metabolic characteristics, such as lipid storage and mobilization [29]. This implies that the observed differences in adipocyte size and number may underlie the distinct lipid metabolic profiles between SAT and PCAT in Yili horses. Smaller and more numerous adipocytes in SAT are typically formed through the differentiation of preadipocytes, which increases adipocyte number and enhances lipid storage capacity, thereby preventing lipid spillover and maintaining systemic insulin sensitivity and metabolic homeostasis. Conversely, the larger adipocytes in PCAT may possess higher lipolytic activity, enabling rapid provision of free fatty acids to adjacent myocardial tissue via paracrine pathways to meet the high energy demands of the heart, and may play specific roles in local cardiovascular regulation and in shaping the inflammatory microenvironment.

In this study, proteomic sequencing was employed to compare the protein expression profiles between SAT and PCAT. The results revealed significant differences in the relative abundance of several proteins, including ACAA2, ENO1, TPI1, PLIN1, COL6A3, and ITGB1, suggesting that these proteins may serve as regulatory factors contributing to the functional heterogeneity of adipose tissues from different anatomical locations. Enrichment analysis indicated that upregulated DEPs were mainly involved in pathways related to glycolysis/gluconeogenesis, oxidative phosphorylation, and fatty acid metabolism, whereas downregulated DEPs were primarily enriched in processes associated with cellular structural maintenance, cell adhesion, and signal transduction. This functional divergence is consistent with the morphological differences observed between adipocytes in SAT and PCAT. In SAT, the enhancement of metabolic pathways may sustain a higher level of lipid turnover, thereby limiting excessive lipid accumulation and restraining adipocyte hypertrophy. In contrast, in PCAT, the enrichment of pathways related to cellular structure and extracellular matrix remodeling may be associated with increased lipid deposition and larger cell size, consistent with the larger adipocyte size observed in this depot. Notably, several key proteins may play important roles in the functional divergence between SAT and PCAT. ACAA2 is a key mitochondrial thiolase involved in the fatty acid β-oxidation pathway, catalyzing the cleavage of long-chain fatty acids to generate acetyl-CoA, thereby supplying energy to the tricarboxylic acid (TCA) cycle and serving as a critical node in the regulation of intracellular lipid homeostasis [30,31]. Numerous studies have demonstrated that the expression level of ACAA2 directly determines the tissue’s capacity to utilize fatty acids. In a chicken embryonic liver model, knockdown of ACAA2 markedly inhibited the expression of PPARα and CPT1, while activating the lipogenic transcription factor SREBP-1c. This led to a significant increase in intracellular triglyceride accumulation and enlarged lipid droplets, indicating that the reduction in ACAA2 expression promotes excessive lipid deposition [32]. In this study, the relative protein abundance of ACAA2 in SAT was significantly higher than that in PCAT, and ACAA2 was significantly enriched in the fatty acid degradation and fatty acid elongation pathways. These results are highly consistent with the transcriptomic findings reported by Wang et al., who identified ACAA2, ACADL, ACOX2, CPT1B, and SLC27A1 as key candidate genes influencing IMF deposition in bovine longissimus dorsi and liver tissues with differing IMF contents. These genes were significantly enriched in pathways related to fatty acid β-oxidation, PPAR signaling, and triglyceride metabolism [33]. Correlation analysis further revealed a highly significant negative association between the relative protein abundance of ACAA2 and adipocyte area and diameter, which aligns well with the functional role of ACAA2 in promoting fatty acid oxidation and preventing excessive triglyceride accumulation. This suggests that ACAA2 may inhibit adipocyte hypertrophy by enhancing lipid catabolism and maintaining a balance between lipid synthesis and degradation, contributing to both morphological and metabolic heterogeneity across distinct adipose depots. In SAT, elevated ACAA2 expression appears to sustain lipid turnover through enhanced fatty acid oxidation, thereby preventing excessive adipocyte enlargement. In contrast, lower ACAA2 expression in PCAT may impede lipid oxidation, forcing incoming fatty acids to be esterified into triglycerides for long-term storage, ultimately leading to markedly increased cell size. Collectively, these findings indicate that ACAA2 likely mediates adipose depot-specific functional heterogeneity by influencing lipid metabolism through fatty acid degradation and elongation pathways. Moreover, six additional differentially expressed proteins, namely ACADVL, ALDH7A1, ECI1, HADHA, HADHB, and MECR, were also significantly enriched in these pathways, indicating the need for further investigation in future studies.

Alpha-enolase (ENO1) is a key enzyme in the glycolytic pathway that, in addition to its role in energy metabolism, also exerts multiple non-glycolytic functions, including regulation of apoptosis and signal transduction [34,35]. Triosephosphate isomerase 1 (TPI1) catalyzes the reversible interconversion between dihydroxyacetone phosphate (DHAP) and glyceraldehyde-3-phosphate (GAP). Because adipocytes lack glycerol kinase, they cannot directly utilize circulating glycerol for triglyceride (TG) synthesis. Instead, they depend on DHAP derived from glycolysis, which can be converted by TPI1 or reduced directly to glycerol-3-phosphate, the essential glycerol backbone required for TG assembly. The expression level of TPI1 therefore directly limits the capacity of adipose tissue to convert glucose into lipid stores. Ge et al. reported that in liver tissues of Chaohu ducks with high and low IMF content, proteins such as ENO1, RGN, TPI1, HSPA9, and PRDX1 regulated lipid metabolism through glycolysis/gluconeogenesis, the pentose phosphate pathway, and fructose and mannose metabolism [36]. Similarly, Shin et al. found that TPI1 expression was significantly higher in the high-marbled group of Hanwoo longissimus dorsi muscles, suggesting that it may promote intramuscular fat deposition by enhancing glycolytic flux [37]. In this study, compared with PCAT, SAT exhibited higher relative protein abundance of ENO1 and TPI1, both of which were significantly enriched in the glycolysis pathway and showed a significant negative correlation with adipocyte area. Glycolysis serves as the principal pathway for energy production and carbon supply in adipocytes, not only generating ATP but also providing glycerol-3-phosphate, a critical precursor for triglyceride synthesis, thereby facilitating lipid droplet formation and fatty acid esterification [38]. As ENO1 and TPI1 are central enzymes within the glycolytic cascade, our findings suggest that SAT in Yili horses possesses a stronger glycolytic capacity and lipid biosynthetic potential, consistent with its predominant role in energy storage.

Perilipin 1 (PLIN1), a member of the PAT protein family, is highly expressed in both white and brown adipose tissues and is predominantly located on the surface of lipid droplets in mature adipocytes [39,40]. As one of the most abundant structural proteins of the lipid droplet phospholipid monolayer, PLIN1 plays a pivotal role in maintaining lipid homeostasis and in regulating lipid droplet formation and lipolysis [41,42]. Previous studies have demonstrated that PLIN1 expression is closely associated with a range of metabolic disorders, including diabetes, liver diseases, and atherosclerosis [43]. Raza et al. [44] found that PLIN1 polymorphisms were significantly correlated with subcutaneous fat thickness, IMF content, thoracic depth, and carcass traits in Qinchuan cattle. In several livestock species, including cattle, sheep, and chickens, PLIN1 has been identified as the most abundant protein in adipose tissue. Overexpression of PLIN1 markedly promotes lipid deposition by significantly upregulating lipogenic genes such as FASN, PPARγ, ACC, LPL, FABP4, DGAT2, and C/EBPβ, while downregulating lipolytic genes PLIN2 and ATGL. Consequently, the number and size of lipid droplets, as well as triglyceride content, are substantially increased, indicating that PLIN1 plays a key role in enhancing lipid accumulation in preadipocytes [45,46,47]. Collectively, these findings indicate that PLIN1 is preferentially expressed in adipose tissue and is involved in adipocyte proliferation, differentiation, and lipid secretion in mature adipocytes. In this study, PLIN1 was highly expressed in PCAT, enriched within the thermogenesis pathway, and positively correlated with adipocyte area. While this finding aligns with previous observations of high PLIN1 expression in adipose tissue, it contrasts with reports in ruminants such as cattle and sheep, where PLIN1 is predominantly enriched in subcutaneous or tail fat depots. This discrepancy is likely attributable to interspecies differences in adipose metabolism and physiological function. Horses, as monogastric animals, possess distinct adipose tissue differentiation and metabolic profiles compared to ruminants, and their long-term physical activity results in a substantially different cardiac metabolic rate. Consequently, the energy supply pressure in PCAT varies among species, potentially influencing the functional localization and expression level of PLIN1. PCAT is a specialized fat depot adjacent to the heart, possessing dual metabolic properties of lipid storage and lipolysis/fatty acid oxidation for energy [48]. Activation of the thermogenesis pathway enhances fatty acid oxidation, prevents excessive lipid droplet accumulation, and supports myocardial energy demands through fatty acid oxidation and local heat release, thereby maintaining cardiac metabolic and thermal homeostasis [49]. Moreover, high PLIN1 expression may help PCAT cope with oxidative stress induced by high oxygen tension and mechanical load. By reinforcing the lipid droplet membrane structure and reducing lipid peroxidation, PLIN1 contributes to cellular lipid homeostasis and prevents lipotoxic responses triggered by excessive lipolysis [50].

The extracellular matrix (ECM), a key structural component of tissues, maintains the stability and plasticity of adipose tissue through interactions with cell membrane receptors, providing both physical support and mechanical signal transduction that regulate adipocyte differentiation, migration, and metabolic activity [51]. Studies have shown that in obesity or metabolic disorders, excessive ECM component deposition (such as collagen) in adipose tissue can induce adipocyte necrosis, activate pro-inflammatory macrophages, and lead to tissue inflammation and metabolic dysfunction [52]. Collagen VI (COL6) is one of the most abundant structural collagens in adipose tissue and is encoded by the genes COL6A1, COL6A2, and COL6A3 [53]. In studies conducted on mice, knockdown of COL6A3 significantly alleviated adipose tissue fibrosis, reduced local inflammation, and improved lipid and glucose metabolism [54]. Stephane Gesta and colleagues found that knockdown of COL6A3 in preadipocytes promoted triglyceride accumulation and lipolysis, increased insulin-induced Akt phosphorylation, and upregulated the expression of adipogenesis-related genes such as PPARγ, GLUT4, ADIPOQ, and FABP4, suggesting that downregulation of COL6A3 enhances adipocyte function and insulin sensitivity [55]. Cui et al. found that in chickens with high IMF content, TIMP2 was significantly upregulated and co-expressed with several lipid metabolism-related proteins, such as SCD, ELOVL7, LPL, as well as COL1A2 and COL6A3, suggesting that TIMP2 can promote fat deposition in muscle tissue through the ECM–receptor interaction signaling pathway [56]. In this study, COL6A3 was highly expressed in SAT and lowly expressed in PCAT. It was significantly enriched in the ECM–receptor interaction pathway, indicating that the lipid metabolic capacity and insulin sensitivity of PCAT are superior to those of SAT, and the ECM–receptor interaction pathway plays a role in regulating lipid metabolism. A highly significant negative correlation was observed between COL6A3 and adipocyte area, which may reflect an increased expression of COL6A3 during tissue remodeling or stress defense to strengthen ECM support and prevent structural damage during adipocyte expansion. Integrin β1 (ITGB1) exhibited an opposite expression pattern. As one of the most widely expressed β subunits of the integrin family, ITGB1 is a typical marker of cell adhesion [57]. Zhang et al. conducted transcriptomic analysis on baNCSCs at different differentiation stages and found that downregulation of ITGB1, KRAS, CCND1, ACTB, VEGFA, MET, and HRAS in the early differentiation stages enhanced adipocyte differentiation [58]. Yu et al. found that ITGB1 is a key regulatory factor influencing intramuscular fat deposition in Qinchuan, Nanyang, and Japanese Black cattle through transcriptomic sequencing [59]. In this study, the relative protein abundance of ITGB1 was higher in PCAT and significantly enriched in the ECM–receptor interaction pathway, indicating that ECM-integrin signaling exhibits tissue-specific regulation across different adipose depots. ITGB1 was positively correlated with adipocyte area, suggesting that in regions with higher ITGB1 expression, ECM-integrin signaling helps maintain cell morphology and promote metabolic activity, thereby enhancing adipocyte health and functionality.

Based on the above analyses of key DEPs, the observed morphological differences between SAT and PCAT appear to be related to variations in protein expression patterns. Although the total number of identified proteins was similar between the two depots, differences in adipocyte morphology were still observed. This may be explained by differences in protein expression levels rather than protein diversity. In particular, proteins involved in lipid metabolism and tissue remodeling processes, including extracellular matrix organization, are likely associated with the structural differences observed between the two adipose depots.

Several limitations of this study should be considered. First, the present work primarily focused on the association between adipose tissue morphology and differentially expressed proteins, without functional validation of the identified candidates. Therefore, the causal roles of these proteins in regulating adipose tissue morphology remain unclear and warrant further investigation. In addition, only male horses were included in this study, which may limit the generalizability of the findings. Future studies incorporating female horses will help provide a more comprehensive understanding and allow assessment of potential sex-related differences in adipose tissue characteristics. Despite these limitations, this study provides a comprehensive comparative analysis of subcutaneous and pericardial adipose tissues in horses at both morphological and proteomic levels. The findings offer important insights into depot-specific adipose biology and highlight candidate proteins and pathways for future functional validation, thereby contributing to a deeper understanding of adipose tissue heterogeneity.

## 5. Conclusions

In summary, this study demonstrates distinct morphological differences between adipose depots in Yili horses: adipocytes in PCAT exhibit larger areas but lower numbers compared to those in SAT. Proteomic analysis identified key candidate proteins, including ACAA2, ENO1, TPI1, PLIN1, COL6A3, and ITGB1. These proteins contribute to the morphological divergence of adipocytes across different depots through their involvement in key biological processes, including lipid metabolism, energy homeostasis, and extracellular matrix remodeling, thereby supporting depot-specific adipose functions. Furthermore, GO and KEGG enrichment analyses indicate that SAT is characterized by more active substrate utilization, energy metabolism, and lipid turnover, whereas PCAT is more associated with structural regulation and cardiovascular-related signaling pathways, highlighting clear depot-specific functional differentiation. These findings not only provide a novel theoretical basis for elucidating the molecular mechanisms underlying adipose tissue heterogeneity in Yili horses but also offer valuable genetic resources for future breeding and genetic improvement programs.

## Data Availability

The data presented in this study are available on request from the corresponding author.

## Abbreviations

The following abbreviations are used in this manuscript:

SAT	Subcutaneous adipose tissue
PCAT	Pericardial adipose tissue
H&E	Hematoxylin and eosin
DEPs	Differentially expressed proteins
GO	Gene Ontology
KEGG	Kyoto Encyclopedia of Genes and Genomes
VAT	Visceral adipose tissue
IMF	Intramuscular fat
PBS	Phosphate-buffered saline
DIA	Data-independent acquisition
DDA	Data-dependent acquisition
FDR	False discovery rate
PPI	Protein–protein interaction
SD	Standard deviation
PCA	Principal component analysis
OPLS-DA	Orthogonal partial least squares discriminant analysis
AAT	Abdominal adipose tissue

## References

[1] Pânzaru C., Doliș M.G., Radu-Rusu R.-M., Pascal C., Maciuc V., Davidescu M.-A. (2024). Equine Milk and Meat: Nutritious and Sustainable Alternatives for Global Food Security and Environmental Sustainability—A Review. Agriculture.

[2] Huang X., He L., Ma J., Li Y., Li J., Zang C., Hou M., Li X. (2025). Ellagic acid on milk production performance, blood and milk hormones, antioxidant capacity and fecal microbial communities in lactating Yili mares. Front. Microbiol..

[3] Lorenzo J.M., Sarriés M.V., Tateo A., Polidori P., Franco D., Lanza M. (2014). Carcass characteristics, meat quality and nutritional value of horsemeat: A review. Meat Sci..

[4] López-Pedrouso M., Lorenzo J.M., Cittadini A., Sarries M.V., Gagaoua M., Franco D. (2023). A proteomic approach to identify biomarkers of foal meat quality: A focus on tenderness, color and intramuscular fat traits. Food Chem..

[5] Lamy A., Costa S., Vial C., Badji I., Carrère M., Rollet P., Amiot M.J. (2023). Horsemeat consumption in France: Determinants and sustainable market perspectives. Meat Sci..

[6] Schumacher M., DelCurto-Wyffels H., Thomson J., Boles J. (2022). Fat Deposition and Fat Effects on Meat Quality-A Review. Animals.

[7] Han Q., Huang X., He J., Zeng Y., Yin J., Yin Y. (2025). Intramuscular fat deposition in pig: A key target for improving pork quality. J. Integr. Agric..

[8] Auger C., Kajimura S. (2023). Adipose Tissue Remodeling in Pathophysiology. Annu. Rev. Pathol..

[9] Wang Y., Li J., Lu D., Meng Q., Song N., Zhou H., Xiao X., Sun L., Zhu H. (2022). Integrated proteome and phosphoproteome analysis of interscapular brown adipose and subcutaneous white adipose tissues upon high fat diet feeding in mouse. J. Proteom..

[10] Contreras G.A., Strieder-Barboza C., De Koster J. (2018). Symposium review: Modulating adipose tissue lipolysis and remodeling to improve immune function during the transition period and early lactation of dairy cows. J. Dairy Sci..

[11] Xu J., Wu T., Lam S.M., Shui G., Yang S., Wang Y., Tao C. (2024). Heterogeneity of Intramuscular, Intermuscular, and Subcutaneous Fat in Laiwu Pigs: Insights from Targeted Lipidomics and Transcriptomics. Agriculture.

[12] Yang X., Zhu R., Song Z., Shi D., Huang J. (2023). Diversity in Cell Morphology, Composition, and Function among Adipose Depots in River Buffaloes. Int. J. Mol. Sci..

[13] Ueda S., Hosoda M., Yoshino K.I., Yamanoue M., Shirai Y. (2021). Gene Expression Analysis Provides New Insights into the Mechanism of Intramuscular Fat Formation in Japanese Black Cattle. Genes.

[14] Jiang G., Shao J., Tang T., Wang M., Wang J., Jia X., Lai S. (2023). TMT-Based Proteomics Analysis Revealed the Protein Changes in Perirenal Fat from Obese Rabbits. Int. J. Mol. Sci..

[15] Wu Z., Wang Z., Wang P., Cheng L., Li J., Luo Y., Yang L., Li L., Zeng J., Hu B. (2024). Integrative analysis of proteomics and lipidomic profiles reveal the fat deposition and meat quality in Duroc × Guangdong small spotted pig. Front. Vet. Sci..

[16] Du Y., Wang Y., Xu Q., Zhu J., Lin Y. (2021). TMT-based quantitative proteomics analysis reveals the key proteins related with the differentiation process of goat intramuscular adipocytes. BMC Genom..

[17] Li X., Meng C., Xue Y., Shen Z., Ren W., Zeng Y., Meng J. (2025). Differential Energy Metabolism in Skeletal Muscle Tissues of Yili Horses Based on Targeted Metabolomics and Transcriptomics Analysis. Biology.

[18] Neeland I.J., Poirier P., Després J.P. (2018). Cardiovascular and Metabolic Heterogeneity of Obesity: Clinical Challenges and Implications for Management. Circulation.

[19] Yang L., Shen Z., Song L., Lu Z., Zeng Y., Wang J., Ren W., Yao X., Meng J. (2026). Integrated targeted metabolomics and transcriptomics analysis reveals heterogeneity of subcutaneous and pericardial adipose tissues in Yili horses. Food Chem..

[20] Koussounadis A., Langdon S.P., Um I.H., Harrison D.J., Smith V.A. (2015). Relationship between differentially expressed mRNA and mRNA-protein correlations in a xenograft model system. Sci. Rep..

[21] Li S., Cao Q., Xiao W., Guo Y., Yang Y., Duan X., Shui W. (2017). Optimization of Acquisition and Data-Processing Parameters for Improved Proteomic Quantification by Sequential Window Acquisition of All Theoretical Fragment Ion Mass Spectrometry. J. Proteome Res..

[22] Wiśniewski J.R., Zougman A., Nagaraj N., Mann M. (2009). Universal sample preparation method for proteome analysis. Nat. Methods.

[23] Maikaew P., Phattanakiatsakul T., Sitticharoon C., Keadkraichaiwat I. (2025). Distinct metabolic associations of subcutaneous and visceral adipocyte morphology in women with or without obesity. Sci. Rep..

[24] Tandon P., Wafer R., Minchin J.E.N. (2018). Adipose morphology and metabolic disease. J. Exp. Biol..

[25] Fostier L., Dauger A., Yvinec R., Ribot M., Audebert C., Soula H. (2026). Rapid cell turnover to model adipocyte size distribution. J. Theor. Biol..

[26] Chait A., den Hartigh L.J. (2020). Adipose Tissue Distribution, Inflammation and Its Metabolic Consequences, Including Diabetes and Cardiovascular Disease. Front. Cardiovasc. Med..

[27] Wang R., Ren W., Li L., Li Z., Ma S., Shan D., Huang Q., Su Y., Wang J. (2025). The characterization of adipose tissue in distinct anatomical regions of the Kazakh horse: A comprehensive analysis of the morphology, fatty acid profile, and lipidomic profile. Front. Anim. Sci..

[28] Depreester E., De Koster J., Van Poucke M., Hostens M., Van den Broeck W., Peelman L., Contreras G.A., Opsomer G. (2018). Influence of adipocyte size and adipose depot on the number of adipose tissue macrophages and the expression of adipokines in dairy cows at the end of pregnancy. J. Dairy Sci..

[29] Akter S.H., Häussler S., Dänicke S., Müller U., von Soosten D., Rehage J., Sauerwein H. (2011). Physiological and conjugated linoleic acid-induced changes of adipocyte size in different fat depots of dairy cows during early lactation. J. Dairy Sci..

[30] Lei X., Xu Q., Li C., Niu B., Ming Y., Li J., Tang Y., Li X., Tang J., Wu J. (2022). Egr1 confers protection against acetaminophen-induced hepatotoxicity via transcriptional upregulating of Acaa2. Int. J. Biol. Sci..

[31] Miltiadou D., Hager-Theodorides A.L., Symeou S., Constantinou C., Psifidi A., Banos G., Tzamaloukas O. (2017). Variants in the 3′ untranslated region of the ovine acetyl-coenzyme A acyltransferase 2 gene are associated with dairy traits and exhibit differential allelic expression. J. Dairy Sci..

[32] Peng M., Kan L., Zhao X., Liu C., Hu W., Tang Y., Meng Y., Sun M., Wang J., Fang F. (2025). Acetyl-CoA acyltransferase 2 as a metabolic modulator: Unraveling its impact on hepatic lipid dynamics in chicken embryos. Biochim. Biophys. Acta Mol. Cell Biol. Lipids.

[33] Wang S., Liu T., Peng P., Fu Y., Shi S., Liang S., Chen X., Wang K., Zhou R. (2025). Integrated Transcriptomic Analysis of Liver and Muscle Tissues Reveals Candidate Genes and Pathways Regulating Intramuscular Fat Deposition in Beef Cattle. Animals.

[34] Shen N., Wang J., Liao J., Yu H., Sun W., Jia X., Lai S. (2025). ENO1 Regulates Apoptosis Induced by Acute Cold Stress in Bovine Mammary Epithelial Cells. Animals.

[35] Aziguli T., Xiao S.Y., Yang Y., Mutailifu M., Li X.Q., Yin S.Q., Ma H.T., Yao H.F., Yao L.L., Hu L.P. (2025). ENO1 promotes PDAC progression by inhibiting CD8(+) T cell infiltration through upregulating PD-L1 expression via HIF-1α signaling. Transl. Oncol..

[36] Ge K., Geng Z. (2022). Proteomic analysis of the liver regulating lipid metabolism in Chaohu ducks using two-dimensional electrophoresis. Open Life Sci..

[37] Shin S.C., Chung E.R. (2016). Identification of differentially expressed genes between high and low marbling score grades of the longissimus lumborum muscle in Hanwoo (Korean cattle). Meat Sci..

[38] Kierans S.J., Taylor C.T. (2024). Glycolysis: A multifaceted metabolic pathway and signaling hub. J. Biol. Chem..

[39] Luo K., Zhuang K., Wu H., Chen Y., Liu Y., Yang F., Wang Z. (2025). PLIN1 suppresses glioma progression through regulating lipid metabolism. Cell Death Dis..

[40] Koshiishi Y., Takahashi R., Murata-Okubo M., Kameyama Y., Souma K., Hirayama H., Wada K. (2024). A PLIN1 polymorphism is associated with fat production in male emus. Poult. Sci..

[41] Maurizi G., Petäistö T., Maurizi A., Della Guardia L. (2018). Key-genes regulating the liposecretion process of mature adipocytes. J. Cell. Physiol..

[42] Renne M.F., Hariri H. (2021). Lipid Droplet-Organelle Contact Sites as Hubs for Fatty Acid Metabolism, Trafficking, and Metabolic Channeling. Front. Cell Dev. Biol..

[43] Gianazza E., Papaianni G.G., Brocca L., Banfi C., Mallia A. (2025). Omics Approaches to Study Perilipins and Their Significant Biological Role in Cardiometabolic Disorders. Int. J. Mol. Sci..

[44] Raza S.H.A., Shijun L., Khan R., Schreurs N.M., Manzari Z., Abd El-Aziz A.H., Ullah I., Kaster N., Shah M.A., Zan L. (2020). Polymorphism of the PLIN1 gene and its association with body measures and ultrasound carcass traits in Qinchuan beef cattle. Genome.

[45] Zhai G., Pang Y., Zou Y., Wang X., Liu J., Zhang Q., Cao Z., Wang N., Li H., Wang Y. (2022). Effects of PLIN1 Gene Knockout on the Proliferation, Apoptosis, Differentiation and Lipolysis of Chicken Preadipocytes. Animals.

[46] Ma Z., Wang W., Zhang D., Zhang Y., Zhao Y., Li X., Zhao L., Cheng J., Xu D., Yang X. (2024). Polymorphisms of PLIN1 and MOGAT1 genes and their association with feed efficiency in Hu sheep. Gene.

[47] Li S., Raza S.H.A., Zhao C., Cheng G., Zan L. (2020). Overexpression of PLIN1 Promotes Lipid Metabolism in Bovine Adipocytes. Animals.

[48] Iacobellis G., Bianco A.C. (2011). Epicardial adipose tissue: Emerging physiological, pathophysiological and clinical features. Trends Endocrinol. Metab..

[49] Sacks H.S., Fain J.N. (2007). Human epicardial adipose tissue: A review. Am. Heart J..

[50] Kimmel A.R., Sztalryd C. (2016). The Perilipins: Major Cytosolic Lipid Droplet-Associated Proteins and Their Roles in Cellular Lipid Storage, Mobilization, and Systemic Homeostasis. Annu. Rev. Nutr..

[51] Ruiz-Ojeda F.J., Méndez-Gutiérrez A., Aguilera C.M., Plaza-Díaz J. (2019). Extracellular Matrix Remodeling of Adipose Tissue in Obesity and Metabolic Diseases. Int. J. Mol. Sci..

[52] Lin D., Chun T.H., Kang L. (2016). Adipose extracellular matrix remodelling in obesity and insulin resistance. Biochem. Pharmacol..

[53] Oh J., Kim C.S., Kim M., Jo W., Sung Y.H., Park J. (2021). Type VI collagen and its cleavage product, endotrophin, cooperatively regulate the adipogenic and lipolytic capacity of adipocytes. Metabolism.

[54] Dankel S.N., Svärd J., Matthä S., Claussnitzer M., Klöting N., Glunk V., Fandalyuk Z., Grytten E., Solsvik M.H., Nielsen H.J. (2014). COL6A3 expression in adipocytes associates with insulin resistance and depends on PPARγ and adipocyte size. Obesity.

[55] Gesta S., Guntur K., Majumdar I.D., Akella S., Vishnudas V.K., Sarangarajan R., Narain N.R. (2016). Reduced expression of collagen VI alpha 3 (COL6A3) confers resistance to inflammation-induced MCP1 expression in adipocytes. Obesity.

[56] Cui H.-X., Luo N., Guo L.-P., Liu L., Xing S.-Y., Zhao G.-P., Wen J. (2023). TIMP2 promotes intramuscular fat deposition by regulating the extracellular matrix in chicken. J. Integr. Agric..

[57] Fang Y., Wu Y., Liu L., Wang H. (2022). The Four Key Genes Participated in and Maintained Atrial Fibrillation Process via Reprogramming Lipid Metabolism in AF Patients. Front. Genet..

[58] Zhang K., Tang X., Zhao R., Yan Y., Song X. (2025). Transcriptomics Analysis of the Adipogenic Differentiation Mechanism of Bovine Adipose-Derived Neural Crest Stem Cells. Animals.

[59] Yu H., Yu S., Guo J., Wang J., Mei C., Abbas Raza S.H., Cheng G., Zan L. (2024). Comprehensive Analysis of Transcriptome and Metabolome Reveals Regulatory Mechanism of Intramuscular Fat Content in Beef Cattle. J. Agric. Food. Chem..

